# Near-Peer Training: Impact of a Single Session on Students’ OSCE Performance

**DOI:** 10.2147/AMEP.S535389

**Published:** 2025-08-22

**Authors:** Andre S Alves, Victor Taramarcaz, Bernard Cerutti, Stephane Genevay, Eduardo Schiffer, Noelle Junod Perron

**Affiliations:** 1 Unit of Development and Research in Medical Education, Faculty of Medicine, Geneva, Switzerland; 2 Department of Medicine, Faculty of Medicine and Geneva University Hospitals, Geneva, Switzerland; 3 Department of Anesthesiology, Pharmacology, Intensive Care Unit and Emergency, Faculty of Medicine and Geneva University Hospitals, Geneva, Switzerland; 4 Medical Directory, Geneva University Hospitals, Geneva, Switzerland

**Keywords:** near-peer, OSCE, transfer, medical students, clinical skills

## Abstract

**Purpose:**

Near-Peer Training (NPT) is increasingly used to teach clinical and procedural skills during undergraduate medical education. The impact of NPT programs on clinical practice is usually measured through OSCE stations that assess the trained skills. Little is known about the impact of a single NPT session on students’ performance in OSCEs. This study aims to assess the impact of a single clinical skill NPT session on students’ overall objective performance at a summative OSCE.

**Patients and Methods:**

This prospective study evaluated the impact of a two-hour NPT session focused on three system-related clinical situations on overall clinical performance. Third-year medical students (junior) practiced these specific clinical skills under the supervision of fourth–sixth year student tutors with students rotating roles as clinician, observer, or patient. Scores (0–100) at the 3^rd^ year summative OSCE served as indicators of objective performance.

**Results:**

In 2022 and 2023, 210 out of 325 junior students underwent the NPT, with 50 tutors recruited for instruction. NPT participants significantly outperformed non-participants in the summative OSCE, with higher mean scores in global assessment (80.01± 7.64 vs 74.58± 6.71, p<0.0001), communication (83.39± 8.99 vs 79.70±10.10, p=0.0011), medical history taking (77.31± 8.93 vs 73.28±9.59, p=0.0006), and physical examination (73.52±10.66 vs 68.30±10.37, p<0.0001). However, there was no evidence of specific performance improvement in OSCE stations related to the trained system related clinical situations (effect of −0.0119±0.0598 on the normalized scores; p=0.8428).

**Conclusion:**

A single NPT session improved junior students’ general performance but not scores related to the trained clinical situations at a summative OSCE. More research is warranted to understand what really boosts student learning since NPT seems to be effective, independently of the clinical skills specifically trained.

## Introduction

Peer-assisted learning, defined as “people from similar social groupings who are not professional teachings, helping each other to learn and learning themselves by teaching”, has been used across all levels of health care education for centuries.^1^ It can be categorized based on group size, more specifically the student-to-student ratio (mentoring, tutoring, or didactic) and the relationship between students: either peer teaching that occurs between students of same levels of education, or near peer teaching (NPT) which takes place in a face-to-face setting with students who are at least one academic year apart.^2^

NPT is increasingly used in undergraduate health professional programs as it is beneficial for health training institutions, students as teachers and students as learners. At the institutional level, it represents a solution to the rising student numbers and a shortage of faculty tutors and mentors in constrained educational environments.^3^,^4^ Regarding seniors students, several studies have shown that they not only improve their clinical knowledge and competences but also develop teaching and leadership skills.^5–7^ Consequently, NPT is now formally recognized worldwide and has led to the development of “medical student-as-teachers” programs in several institutions to support students in acquiring teaching skills.^8^,^9^ Junior students also benefit from NPT, often achieving comparable or even superior learning outcomes compared to traditional teaching methods.^10^,^11^ NPT has proven particularly effective during the clinical years and when the content focuses on practical and procedural clinical skills.^10^,^12^

NPT often targets clinical skills including history taking, physical exam, communication, or procedural skills such as suturing, ultrasound or basic life support. Studies evaluating the impact of NPT programs through objective structured clinical encounters (OSCE) have shown that most NPT programs support learning over time and promote consistent improvements in OSCE performance.^10^,^11^ However, little is known about the effectiveness of a single NPT training delivered alongside traditional faculty-led clinical skills training. This study aimed to assess the impact of a single NPT session on students’ overall performance in a summative OSCE.

## Method

### Setting and Design

We developed and evaluated the impact of a new NPT session on third year medical students’ clinical performance at the Faculty of Medicine, Geneva University, Switzerland. The Geneva Faculty of Medicine offers a six-year curriculum divided into three pre-clinical years (bachelor’ level, 480 students) and three clinical years (master’s level, 480 students) with approximately 160 students per year. Clinical skills training takes place during the second and third years of the bachelor. During these two years, medical students practice history taking, physical examination, and communication skills, through four formative OSCEs, each focusing on a different system: abdominal, cardiac, respiratory, and neurological. Two formats are used: 1) a group format involving direct observation followed by immediate feedback. One clinical teacher supervises two or three students interacting consecutively with a standardized patient mimicking a different clinical problem, followed by group feedback from the teacher, peers, and simulated patient; 2) an individualised video-based format—where students receive delayed verbal feedback given by a clinical teacher after a videotaped encounter with a standardized patient. At the end of the third year of the bachelor, students take a summative OSCE of three stations, covering topics such as abdominal, cardiac, respiratory, musculoskeletal, neurological, gynaecological, emergency, and hematologic related conditions.

### Development of the Near-Peer Clinical Skills Training

It consisted of a two-hour session during which all third-year students (junior students) could rehearse and improve clinical skills on three clinical situations. The clinical situations were related to systems for which students from previous years had shown weaker history taking and physical examination skills than in other systems at the end of the bachelor years. The first two clinical situations focused on musculoskeletal and neurological complaints for all students, and the third addressed gynaecological, emergency, or hematologic related issues. The tutor facilitated a group of three students successively role-playing the clinician, the observer, or the patient. The observer’s role was to provide feedback on the clinician’s history-taking and physical examination skills—using a grid—prior to the facilitator’s input. The clinical situations were developed by experienced clinical teachers and aligned with previously taught clinical skills. The NPT was limited to one session for feasibility and was optional, due the limited availability of both junior and senior students.

### Participants

As part of a prospective study held in 2022 and 2023, near-peer teachers—fourth to sixth year medical students (senior students: approximately 160 per year)—were invited by Email to attend this optional NPT session. Fifty of them (n=26 in 2022, and n=24 in 2023) accepted the invitation and attended a two-hour session. This session included an overview of the learning objectives, content, process and organisation of the training, and their role. The second part consisted of a 90-min small group training session in which senior students practiced clinical and teaching skills such as feedback and small group facilitation. Senior students alternated roles as supervisor, the clinician interviewing the patient, or the observer during 3–5 min sequences. These sessions were led by two senior students (ASA and VT) in charge of the project. Participants then registered online to facilitate one or two NPT sessions focused on clinical skills training.

All junior (third year) medical students were invited to attend this optional NPT session via Email over two consecutive years: 246 registered (122 out of 160 in 2022 and 124 out of 160 in 2023) and 210 attended the training session (114 in 2022 and 96 in 2023).

### Outcome Measures

We collected junior students overall score and sub-scores regarding history taking, physical exam, and communication skills at the end of the third year summative OSCE exam. Each of the three stations of the exam lasted 18 minutes—and assessed history taking, physical exam, and communication skills.

The study project was approved by the Ethics Committee of the Geneva University, Geneva Switzerland (CUREG-2023-03-50). All junior and senior students provided written consent for the use of their data.

### Analysis

A multiple regression model was used to investigate the potential association of the OSCE scores (raw scores, ie number of points divided by the maximum of attributable points of the evaluation grid) and the following categorical variables: gender (gender influences performance in some clinical skills such as communication^13^), set of OSCEs stations used the day of the exam, and participation in the NPT.

Additional complementary analysis used the same model with two additional variables: the scores from the two formative OSCEs taken during the third year. These two variables were considered as student’s baseline performance prior to the NPT and summative OSCE.

Finally, a linear mixed effect model was used to investigate whether there was a link between the performance at every single station of the OSCE and the fact that this station had dealt with a system specifically trained during the NPT. All the OSCE station scores were normalized and taken into the model as with the following variables: gender, participation in the NPT, system specifically trained during the NPT (fixed effects), and individual-specific effect (random). The validity of the models was checked by visual inspection of the plot and quantile–quantile normal plot of the residuals.

All analyses were run on R 4.4.2 (the R Foundation for Statistical Computing, Vienna, Austria).

## Results

**Figure 1:**
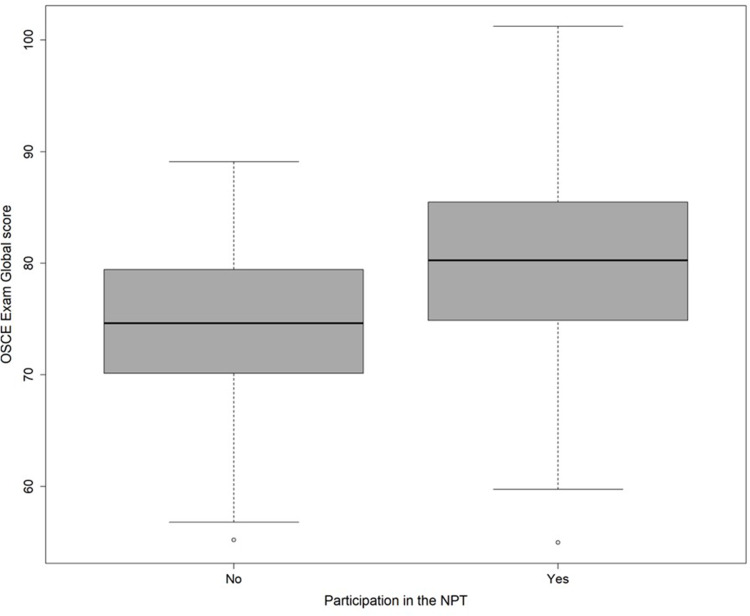
Boxplot of the OSCE global scores according to near-peer OSCE participation (yes; n=208) or nonparticipation (no; n=111).

Three hundred and nineteen students (153 in 2022 and 166 in 2023) attended the end of third year summative OSCE (57% women and 43% male). Junior medical students who participated in a near-peer teaching program significantly outperformed non-participants in the summative OSCE, independently from other variables such as gender, scores at prior third year formative OSCE, topics of the summative OSCE and across all the dimensions assessed (global score 80.01±7.64 vs 74.58±6.71 p-value < 0.0001) (Table 1 and Figure 1).

**Table 1:** Third Year Medical Students’ Scores at the Summative OSCE Whether They Took Part in the NPT Study or Not

	NPT Participation Score (Mean±SD) n=208	NPT Nonparticipation Score (Mean±SD) n=111	p-value*	p-value**
Global	80.01±7.64	74.58±6.71	<0.0001	<0.0001
History taking	77.31±8.93	73.28±9.59	0.0006	0.0003
Physical exam	73.52±10.66	68.30±10.37	<0.0001	0.0112
Communication	83.39±8.99	79.70±10.10	0.0011	0.0581

**Notes**: *The p-values are derived from a multiple regression model with the following additional covariates: gender, set of OSCEs stations used the day of the exam. ** and in addition, for the right-side column, the scores at the two formative OSCE previously held during the same academic year.

Performance at prior third year formative OSCEs was not different between NPT attenders and non-attenders (81.58±8.74 vs 80.35±9.13; p-value=0.6345), and integration of these results in the model led to the same conclusion regarding the difference between the NPT and the other group, apart from weaker evidence for the communication subscale.

Further sub-analysis showed that students who attended the NPT did not systematically obtain higher scores in the OSCE stations specifically related to the clinical situations for which they received additional clinical skills training (musculoskeletal-hip global score 66.70±9.89 vs 67.49±9.89 p-value 0.820; neurology global score 75.42±8.34 vs 75.94±8.03 p-value 0.695; lymphatic global score 77.17±9.67 vs 75.20±8.79 p-value 0.598 in 2022) (musculoskeletal global score 84.85±11.42 vs 77.21±12.59 p-value 0.013; neurology global score 72.44±11.2 vs 62.44±11.52 p-value 0.018; emergency global score 66.71±16.57 vs 57.74 ±15.14 p-value 0.067 in 2023) (Appendix 1).

The linear mixed effect model used to investigate whether there was a link between the performances at every single OSCE station of the exam confirmed a strong effect of the participation in the NPT (0.4075±0.0771; p<0.0001). There was, however, no evidence of any additional benefit specifically linked to the station focused on a system specifically trained during the NPT (−0.0119±0.0598; p=0.8428).

## Discussion

This study aimed to assess the impact of a single NPT session on students’ overall objective performance in a summative OSCE. We were especially interested in evaluating whether students’ performance was higher for the systems specifically trained during the NPT. We showed that students who attended this new near peer led clinical skills training session obtained higher grades at the summative OSCE than non-participating students during two consecutive years, independently from other factors such as gender, scores at prior 3^rd^ year formative OSCEs, topics of the summative OSCE. The effect extended to all the dimensions of the OSCE (history taking, physical examination, and communication) and was independent from the clinical situations trained during the NPT session.

Improved performance at summative OSCEs may be explained by the fact that participation was optional, and that only highly motivated and already skilled students may have attended such additional training activity.^14–16^ However, several studies have shown that higher grades are more associated with peer-facilitated sessions than other factors such as previous academic grades.^17^,^18^ In our study, the absence of significant differences in prior formative OSCE scores between participants and non-participants suggests that the two groups were comparable in terms of baseline performance. Furthermore, we also found that medical students who attended this single NPT did not consistently outperform non-attenders in OSCE stations related to the clinical scenarios covered during the NPT session. Several studies have reported that peer-teachers are preferred to medical teachers for various reasons: senior students are familiar with the exam content, have completed the same curriculum and can share their own experience and highlight common pitfalls; finally, despite being less clinically experienced than Faculty educators, they deliver information relevant to the expected level.^19–21^ In addition, working cooperatively, in a secure learning environment can empower students’ learning and increase their self-confidence by decreasing their anxiety and the stress related to the upcoming exam.^22^,^23^ Similarly, peer-led learning in clinical environments has been shown to positively influence and support medical students’ clinical development during clerkships.^24^ These elements, which refer to cognitive and social congruence may explain why this single NPT was effective despite its short duration and its limited focus. Cognitive congruence refers to the fact that as near-peer tutors are usually only one or two years apart, this makes it easier for them to identify students’ needs, share past experiences and give useful advice.^12^,^25^ The concept of social congruence concerns senior and junior students sharing similar roles.^16^ Being a student helps build a rapport with students that goes beyond the traditional teacher-student dynamic and creates a more collaborative and mutually respectful interaction.^7^ A recent study evaluating the effectiveness of near-peer teaching (NPT) among third-year medical students found that participants reported an improved understanding of how their clinical skills would be assessed during OSCEs.^26^ This further confirms that NPT not only enhances clinical skill acquisition but also provides valuable insights and strategies for preparing for OSCEs. This may explain why a single NPT improve student’s performance at OSCEs, independently from the specificity of the skills trained during the session.

There are, however, several limitations. As noted, both senior and junior student recruitment was voluntary and limited to a single institution, raising the possibility of a selection bias toward high-performing students. However, the fact that participation to this NPT was not associated with the level of performance at previous formative OSCE does not support this hypothesis. A randomized trial would have been the best design to test the effectiveness of NPT but was not possible due to the voluntary nature of the intervention. Additionally, no selection criteria were applied to senior students, and we did not collect information about their prior academic performance or teaching experience. As a result, we cannot determine whether these factors influenced the quality of the NPT sessions.

## Conclusion

A single NPT session seems to improve junior students’ general performance but not specifically the scores related to the trained clinical situations at a summative OSCE. This suggests that NPT may facilitate the transfer of more generic rather than specific skills or boost students’ confidence and skill acquisition by providing opportunities to gain additional insight into how to prepare for the OSCE. Further research is needed to better understand the mechanisms that really enhance student learning in such context.

## Data Availability

The datasets generated or analyzed during the current study are not publicly available due to the privacy of the students but are available from the corresponding author on reasonable request.
